# Relationship between estimated pulse wave velocity and the risk of future sarcopenia in middle-aged and older Chinese adults: evidence from the China Health and Retirement Longitudinal Study

**DOI:** 10.3389/fcvm.2025.1494635

**Published:** 2025-02-17

**Authors:** Dingding Song, Jianghu Miao, Yunzhu Zhang, Aiguo Zhu

**Affiliations:** Department of Geriatrics, Shanghai Pudong New Area People’s Hospital, Shanghai, China

**Keywords:** sarcopenia, pulse wave velocity, China Health and Retirement Longitudinal Study, arterial stiffness, middle-aged, older people

## Abstract

**Objective:**

Sarcopenia, common among older adults, is associated with adverse health outcomes. This study explores the relationship between estimated pulse wave velocity (ePWV), a marker of arterial stiffness, and sarcopenia to support the early diagnosis and prevention strategies.

**Methods:**

Using data from two waves (2011 and 2015) of the China Health and Retirement Longitudinal Study (CHARLS), we conducted a nationally representative cohort study. ePWV was calculated at baseline, and sarcopenia was identified based on the Asian Working Group for Sarcopenia (AWGS) 2019 criteria. We used multivariate logistic regression and restricted cubic spline (RCS) analyses to investigate the ePWV-sarcopenia relationship.

**Results:**

Among 6,639 participants followed for 4 years, 11.1% were diagnosed with sarcopenia. Initial analyses indicated a positive correlation between ePWV and sarcopenia (*β* = 1.35), which weakened after adjusting for confounders. RCS analysis demonstrated a non-linear relationship (P-non-linear <0.001), with sarcopenia risk peaking at an ePWV of 9.430 m/s. The highest ePWV quartile showed the lowest grip strength, the longest chair stand test time, and the highest sarcopenia prevalence (*P* < 0.0001).

**Conclusions:**

In middle-aged and elderly Chinese adults, ePWV variations may be linked to sarcopenia risk, potentially serving as a predictive marker. The non-linear relationship indicates complex underlying mechanisms, meriting further research.

## 1 Introduction

As the global population ages, the prevalence of sarcopenia is increasing. Globally, more than 50 million adults over the age of 50 suffer from sarcopenia, and by 2050, this number could reach 500 million (1, 2). Age-related loss of muscle mass is accompanied by reduced muscle strength and/or poor physical performance in sarcopenia (3). Extensive evidence links sarcopenia to various adverse outcomes, including falls, frailty, increased mortality, and higher healthcare utilization, making it a significant public health concern (4–7). Notably, due to the influence of factors such as ethnicity, environment, diet, and physical activity on skeletal muscle, there is still no internationally recognized, unified diagnostic criterion for sarcopenia. However, several organizations have proposed diagnostic guidelines, with the most widely used being the criteria set by the European Working Group on Sarcopenia in Older People (EWGSOP), the International Working Group on Sarcopenia (IWGS), and the Asian Working Group for Sarcopenia (AWGS) (8).

Arterial stiffening is another physiological process closely linked to aging. Increased arterial stiffness significantly raises the incidence and mortality of cardiovascular diseases (CVD) (9). As a result, arterial stiffness is increasingly used as a preclinical indicator of CVD, typically measured through pulse wave velocity (PWV). Currently, carotid-femoral PWV (cfPWV) and brachial-ankle PWV (baPWV) are the two most common PWV measurement methods (10), with cfPWV considered the gold standard for assessing arterial stiffness (11). However, the clinical use of cfPWV and baPWV is limited by their high cost and operational complexity (12, 13). To address these limitations, researchers developed the concept of estimated pulse wave velocity (ePWV) as an alternative method for assessing arterial stiffness. ePWV is calculated using an algorithm that incorporates age and mean blood pressure (MBP) (14). Although ePWV cannot fully replace cfPWV, studies have confirmed that it can reliably reflect the degree of arterial stiffness (15, 16), offering a more convenient and economical assessment method for large-scale population studies.

While the relationship between PWV and cardiovascular outcomes has been extensively studied, its association with non-cardiovascular outcomes such as sarcopenia remains limited. Some studies have begun to explore this area: Research by Sun et al., Rong et al., and Zhang Y. et al. found that subjects with sarcopenia had higher baPWV measurements than those without sarcopenia (17–19). The Health ABC observational cohort analysis discovered that carotid-femoral pulse wave velocity (cfPWV) was associated with skeletal muscle decline (20). A study from China indicated that high brachial-ankle pulse wave velocity in community-dwelling older adults was associated with muscle loss, with gender differences observed (21). However, there is still a notable gap in research regarding ePWV and incident sarcopenia. Particularly in the Chinese population, this relationship has not been thoroughly explored. Given the convenience and cost-effectiveness of ePWV measurement, investigating the relationship between ePWV and the risk of future sarcopenia may provide new insights for large-scale screening and prevention strategies.

Drawing from the previously discussed context, the objective of this research is to: (1) Investigate the association between ePWV and the risk of future sarcopenia among the Chinese demographic. (2) Examine the link between ePWV and indicators related to sarcopenia, including muscle mass, strength, and physical capability. (3) Evaluate the potential of ePWV as a predictor of future sarcopenia. By employing data from the China Health and Retirement Longitudinal Study (CHARLS), this study aims to provide valuable understanding regarding the connection between arterial rigidity and the risk of future sarcopenia, while laying the groundwork for strategies aimed at the early detection and prevention of this condition. This research not only addresses existing gaps in understanding but could also serve as an essential reference for both clinical applications and public health strategies.

## 2 Methods

### 2.1 Study population

The China Health and Retirement Longitudinal Study (CHARLS) is a longitudinal research effort that includes sample of individuals aged 45 and older in China. Its primary objective is to comprehensively explore the aspects of population aging, as well as its diverse influences on public health and economic progress within the country. To achieve this, CHARLS utilized a probability sampling method that is proportional to the size and administered its baseline survey in the year 2011. This initial survey captured data from 17,708 participants belonging to 10,257 households situated in 450 villages or communities across 150 districts and counties within 28 provinces (including autonomous regions and municipalities) throughout China. The survey encompasses an extensive array of subjects such as socio-demographic factors, job status, household wealth, health status, and the medical insurance provisions for the respondents and their families. Moreover, the research involved the collection of blood samples and 13 various physical measurement metrics from the participants, yielding significant data for comprehensive biomedical inquiry. In order to monitor the evolving dynamics of population aging, CHARLS performs follow-up surveys biennially to collect up-to-date information. As of now, CHARLS has effectively conducted and published five data waves (2011, 2013, 2015, 2018, 2020), providing valuable longitudinal insights for examining the phenomenon of population aging in China. Comprehensive details regarding CHARLS can be found in the pertinent academic literature (22). The dataset is accessible for download via the following URL: http://CHARLS.pku.edu.cn/en. Approval for CHARLS was granted by the Biomedical Ethics Review Committee of Peking University (IRB00001052-11015). All individuals participating in the study provided informed consent. Additionally, the research adhered to the guidelines set forth by the Strengthening the Reporting of Observational Studies in Epidemiology (STROBE) (23).

### 2.2 Sarcopenia and related indicators

This research utilized the 2019 consensus criteria established by the Asian Working Group for Sarcopenia (AWGS) to evaluate the status of sarcopenia. These criteria focus on three primary components: muscle strength, appendicular skeletal muscle mass (ASM), and physical performance. Muscle strength assessment was conducted using grip strength measured with a dynamometer. Low muscle strength was indicated by an average grip strength of less than 28 kg for males and below 18 kg for females. The calculation for ASM employed a validated anthropometric equation tailored for the Chinese demographic: ASM = 0.193 × weight (kg) + 0.107 × height (cm) − 4.157 × sex (male = 1, female = 2) − 0.037 × age − 2.631 (3). This equation demonstrated strong alignment with measurements obtained from dual-energy x-ray absorptiometry (DXA). The skeletal muscle mass index (SMI) was determined using the formula SMI = ASM/height^2^ (24). The thresholds for low muscle mass were established at the lowest 20% of SMI based on gender within the study group, which amounted to less than 7.08 kg/m^2^ for males and under 5.38 kg/m^2^ for females (25). Physical performance was evaluated through either gait speed or the five-time chair stand test, where a gait speed of less than 1.0 m/s or a completion time of 12 s or more for the chair stand test indicated diminished physical function.

In accordance with the AWGS 2019 guidelines, the criteria for diagnosing sarcopenia include a reduction in muscle mass, which is accompanied by either diminished muscle strength or a decline in physical function. Those who show concurrent decreases in muscle mass, strength, and physical function have been categorized as suffering from severe sarcopenia. Conversely, individuals displaying only reduced muscle strength, regardless of the presence of decreased physical function, have been considered as possibly having sarcopenia. Due to the limited number of participants identified with severe sarcopenia, this research merged them into the broader sarcopenia category. Ultimately, the study focused on incident sarcopenia, referring to individuals who did not have sarcopenia or were not considered at risk in 2011, but were later diagnosed with sarcopenia by 2015.

### 2.3 Measurement of ePWV

In both the initial and subsequent phases of the research, the CHARLS team employed an Omron HEM-7200 electronic device to assess the blood pressure of participants. During these measurements, individuals were instructed to remain seated, calm, and in a quiet environment. A sequence of three measurements was taken, with the mean value being utilized to determine systolic blood pressure (SBP) and diastolic blood pressure (DBP). The estimated pulse wave velocity (ePWV) was calculated using the equation suggested by Greve et al. (14), derived from the reference values of arterial stiffness collaboration (26). Distinct calculation formulas were applied based on the presence of cardiovascular risk factors, which include smoking, hypertension, dyslipidemia, or diabetes. For those participants identified as having cardiovascular risk factors, the ePWV was calculated using the following formula: 9.587 − 0.402 × age + 4.560 × 10^−3^ × age^2^ − 2.621 × 10^−5^ × age^2^ × MBP + 3.176 × 10^−3^ × age × MBP − 1.832 × 10^−2^ × MBP. Conversely, the formula for participants without cardiovascular risk factors was: 4.62 − 0.13 × age + 0.0018 × age^2^ + 0.0006 × age × MBP + 0.0284 × MBP. In these equations, the mean arterial pressure (MBP) was derived from: DBP + 0.4(SBP − DBP) (15).

### 2.4 Covariates

This research identified a range of baseline variables to serve as covariates, which encompass demographic traits, health-related behaviors, chronic conditions, and biochemical metrics. The variables examined include age, gender, geographical location, educational attainment, marital status, consumption of alcohol and tobacco, duration of nighttime sleep, weight, height, body mass index (BMI), waist circumference, and levels of biomarkers such as C-reactive protein, creatinine, along with HDL and LDL cholesterol. In terms of chronic conditions, we evaluated the occurrence of hypertension, dyslipidemia, diabetes, cancer, liver ailments, chronic respiratory diseases, heart issues, strokes, kidney problems, stomach disorders, and arthritis. Furthermore, the evaluation included an analysis of instrumental activities of daily living (IADL).

The precise criteria for various indicators are defined as follows: Hypertension is characterized by self-reported medical diagnoses or a systolic blood pressure of 140 mmHg or greater, or a diastolic blood pressure of 90 mmHg or higher; dyslipidemia is indicated by LDL cholesterol levels of 3.4 mmol/L or more, or HDL cholesterol levels of less than 1.0 mmol/L (for males) or less than 1.3 mmol/L (for females), or self-reported medical diagnoses; diabetes is identified through self-reported diagnoses, fasting blood glucose of 7 mmol/L or higher, random blood glucose levels of 11.1 mmol/L or more, or glycated hemoglobin levels of 6.5% or greater; other chronic conditions were evaluated based on self-reported medical assessments; IADL was measured using a standardized scale to gauge individuals’ capabilities in conducting daily living activities.

### 2.5 Statistical analysis

For the descriptive analysis, we categorized ePWV into quartiles. Means and standard deviations (SD) were utilized for continuous variables according to their respective distributions. Appropriate statistical techniques were applied when comparing groups of continuous variables. For data that was normally distributed, Student's *t*-test was conducted, whereas the Mann–Whitney *U* test was applied for those that did not follow a normal distribution. Categorical variables were represented as percentages, and depending on the characteristics of the sample size and distribution, between-group comparisons were conducted using either Fisher's exact test or Pearson's chi-square test.

For the association analysis, logistic regression models and ordinary least squares (OLS) models were employed to investigate the relationship between ePWV and the occurrence of sarcopenia, as well as the various assessment indicators of sarcopenia, which include muscle strength, appendicular skeletal muscle mass (ASM), and physical performance. We implemented a range of covariate combinations across four different models. The Crude model did not account for any covariates. In Model 1, adjustments were made for age, gender, BMI, marital status, education level, residence, and sleep duration. Model 2 incorporated adjustments for smoking history, alcohol consumption, IADL, diabetes, chronic liver disease (CLD), hypertension, liver ailments, stroke history, kidney issues, gastrointestinal disorders, dyslipidemia, and arthritis in addition to the variables included in Model 1. Model 3 further included C-reactive protein, creatinine, HDL, and LDL levels on top of the adjustments made in Model 2. Moreover, we utilized restricted cubic splines (RCS) to examine the non-linear relationship between ePWV and sarcopenia, along with its assessment indicators. In order to verify the robustness of the analytical findings, this study also performed subgroup analyses utilizing logistic regression models to explore whether the association between ePWV and sarcopenia varied across different subgroups, including age, gender, marital status, smoking status, diabetes, heart disease, stroke, hypertension, and IADL. This approach aids in elucidating the influence and clinical relevance of ePWV on sarcopenia within diverse populations.

In this study, the proportion of missing values for most variables is less than 5%, and for a small number of variables, it is less than 25%, which meets the conditions for applying the predictive mean matching (PMM) imputation method. Therefore, covariates with missing values (less than 25%), predictive mean matching (PMM) imputation was applied. All statistical analyses were conducted using R Studio (R version 4.41). The hypothesis tests in this investigation were two-tailed, with a threshold of *p*-value lower than 0.05 regarded as statistically significant.

## 3 Results

### 3.1 Baseline characteristics of the study population

**Figure 1:**
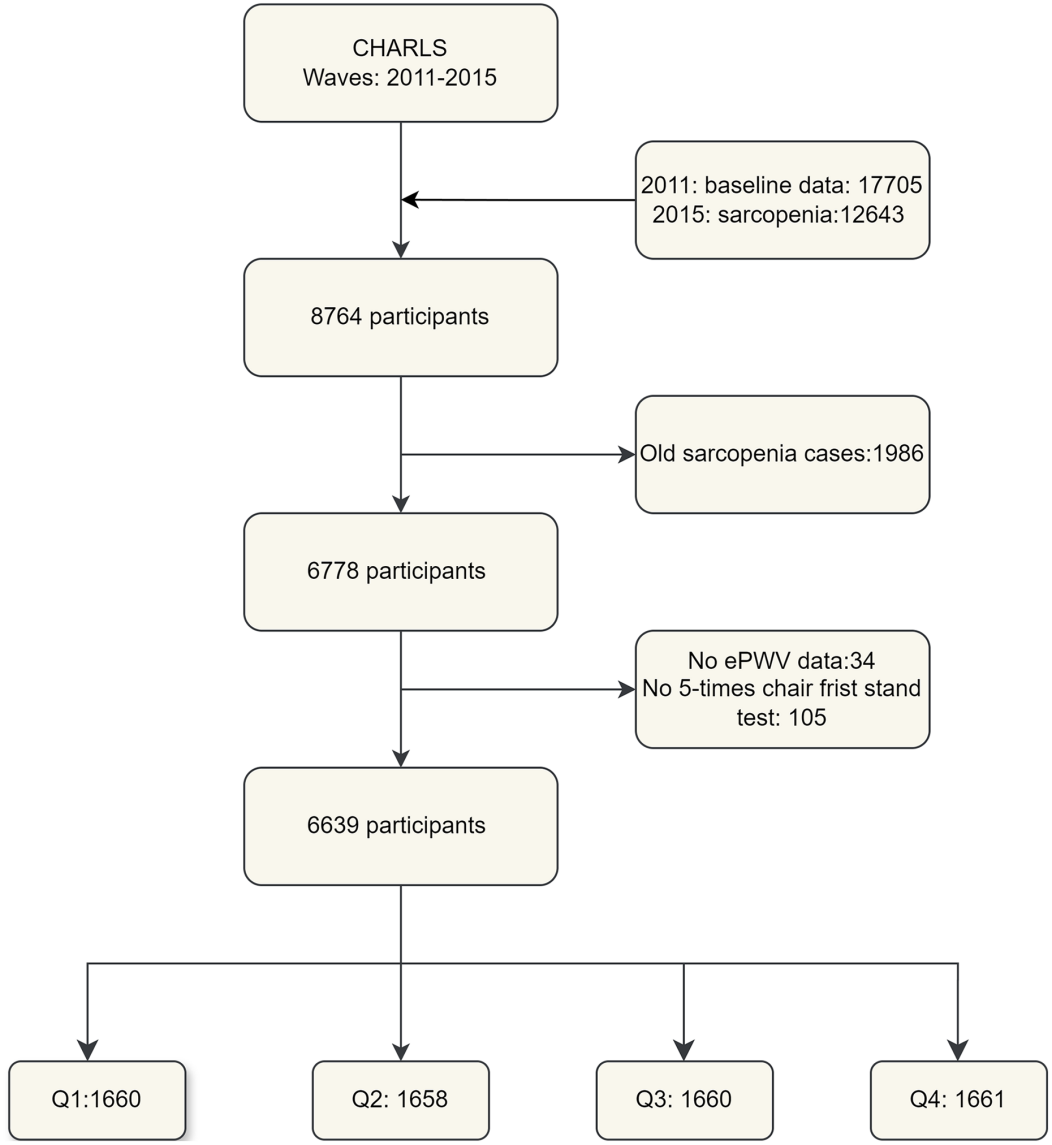
Flowchart illustrating the detailed process of sample selection in the study.

We extracted data from the first wave (2011) and third wave (2015) of the CHARLS database. The first wave included 17,705 participants, while the third wave contained sarcopenia information for 12,643 individuals. After merging these datasets, we obtained a sample of 8,764 participants. We then excluded 1,986 individuals diagnosed with sarcopenia in 2011, as well as those lacking ePWV data or missing the five-time chair stand test, resulting in a final study population of 6,639 participants (Figure 1 provides a detailed flowchart of this process).

We conducted statistical analyses after dividing ePWV into quartiles: Q1 [5.18, 7.906], Q2 (7.906, 8.918], Q3 (8.918, 10.133], and Q4 (10.133, 16.589]. The mean age of the included population was 56.71 years, with an average weight of 60.94 kg and a mean body mass index (BMI) of 24.09 kg/m^2^. The gender distribution was 46.69% male and 53.31% female, with 11.10% diagnosed of participants with sarcopenia. The average grip strength was 31.40 ± 9.53, the five-time chair stand test averaged 9.20 ± 3.24 s, and the mean SMI was 6.91 ± 1.07. Using the lowest quartile (Q1) as a reference, participants in Q4 demonstrated the lowest grip strength levels, required the most time for the five-time chair stand test, and had the highest number of sarcopenia diagnoses. These data suggest that as ePWV values increase, participants’ muscle function and overall health status may show a declining trend. Additional information is presented in Table 1.

**Table 1:** Study population baseline characteristics in 2011.

Variable	Total (*n* = 6,639)	Q1 (*n* = 1,660)	Q2 (*n* = 1,658)	Q3 (*n* = 1,660)	Q4 (*n* = 1,661)	Statistic	*p*.value
ePWV (m/s)	9.12 ± 1.65	7.21 ± 0.50	8.43 ± 0.29	9.48 ± 0.35	11.38 ± 1.04	13,513.25	**<0**.**0001**
Age (year)	56.71 ± 7.79	50.02 ± 4.05	54.05 ± 5.16	58.00 ± 5.63	64.74 ± 7.09	2,090.69	**<0**.**0001**
Sleeping time (h)	6.46 ± 1.78	6.60 ± 1.71	6.53 ± 1.75	6.37 ± 1.77	6.34 ± 1.89	8.11	**<0**.**0001**
Weight (kg)	60.94 ± 10.79	58.87 ± 9.80	61.34 ± 10.92	62.02 ± 10.81	61.52 ± 11.31	28.48	**<0**.**0001**
Height (cm)	158.90 ± 8.18	158.82 ± 7.70	159.56 ± 8.15	159.22 ± 8.34	158.02 ± 8.43	11.01	**<0**.**0001**
BMI (kg/m^2^)	24.09 ± 3.61	23.31 ± 3.31	24.05 ± 3.70	24.42 ± 3.57	24.57 ± 3.73	40.99	**<0**.**0001**
Waist (cm)	85.45 ± 12.08	81.83 ± 11.88	84.99 ± 12.20	86.82 ± 11.36	88.15 ± 11.93	88.49	**<0**.**0001**
Grip (kg)	31.40 ± 9.53	31.88 ± 9.65	32.42 ± 9.83	31.48 ± 9.09	29.80 ± 9.35	23.55	**<0**.**0001**
Five times sit to stand test (s)	9.20 ± 3.24	8.49 ± 2.40	8.96 ± 2.97	9.15 ± 3.07	10.19 ± 4.05	84.64	**<0**.**0001**
SMI (kg/m^2^)	6.91 ± 1.07	6.74 ± 1.00	6.97 ± 1.07	6.99 ± 1.06	6.94 ± 1.12	19.86	**<0**.**0001**
CRP (mg/L)	2.27 ± 6.18	1.87 ± 4.64	2.23 ± 7.41	2.40 ± 6.76	2.57 ± 5.49	3.03	**0**.**03**
Creatinine (mg/dl)	0.77 ± 0.18	0.73 ± 0.15	0.76 ± 0.16	0.78 ± 0.19	0.82 ± 0.21	48.49	**<0**.**0001**
HDL (mg/dl)	50.26 ± 14.65	52.32 ± 14.74	49.96 ± 14.24	49.41 ± 14.69	49.42 ± 14.73	11.39	**<0**.**0001**
LDL (mg/dl)	116.98 ± 34.81	111.87 ± 32.07	116.68 ± 33.74	118.45 ± 36.42	120.73 ± 36.16	14.90	**<0**.**0001**
Sarcopenia (baseline)						46.22	**<0**.**0001**
No	6,254 (94.20)	1,604 (96.63)	1,572 (94.81)	1,563 (94.16)	1,515 (91.21)		
Possible	385 (5.80)	56 (3.37)	86 (5.19)	97 (5.84)	146 (8.79)		
Sarcopenia						147.54	**<0**.**0001**
No	5,902 (88.90)	1,566 (94.34)	1,516 (91.44)	1,462 (88.07)	1,358 (81.76)		
Yes	737 (11.10)	94 (5.66)	142 (8.56)	198 (11.93)	303 (18.24)		
Sex						57.31	**<0**.**0001**
Male	3,100 (46.69)	647 (38.98)	789 (47.59)	814 (49.04)	850 (51.17)		
Female	3,539 (53.31)	1,013 (61.02)	869 (52.41)	846 (50.96)	811 (48.83)		
Marital status						109.56	**<0**.**0001**
Married and living with spouse	5,757 (86.71)	1,481 (89.22)	1,497 (90.29)	1,462 (88.07)	1,317 (79.29)		
Others	882 (13.29)	179 (10.78)	161 (9.71)	198 (11.93)	344 (20.71)		
Education						218.50	**<0**.**0001**
College or above	95 (1.43)	23 (1.39)	20 (1.21)	26 (1.57)	26 (1.57)		
Middle school	2,190 (32.99)	708 (42.65)	643 (38.78)	490 (29.52)	349 (21.02)		
No formal education	2,782 (41.91)	565 (34.04)	655 (39.51)	724 (43.61)	838 (50.48)		
Primary school	1,571 (23.67)	364 (21.93)	340 (20.51)	420 (25.30)	447 (26.93)		
Residence						9.59	**0**.**02**
Rural	4,280 (64.47)	1,101 (66.33)	1,074 (64.78)	1,084 (65.30)	1,021 (61.47)		
Urban	2,359 (35.53)	559 (33.67)	584 (35.22)	576 (34.70)	640 (38.53)		
Smoke						13.75	**<0**.**01**
No	4,625 (69.71)	1,213 (73.12)	1,118 (67.47)	1,151 (69.42)	1,143 (68.81)		
Yes	2,010 (30.29)	446 (26.88)	539 (32.53)	507 (30.58)	518 (31.19)		
Drink						10.67	**0**.**01**
No	4,379 (66.00)	1,128 (67.99)	1,045 (63.07)	1,088 (65.62)	1,118 (67.31)		
Yes	2,256 (34.00)	531 (32.01)	612 (36.93)	570 (34.38)	543 (32.69)		
IADL						53.01	**<0**.**0001**
No	6,074 (91.54)	1,567 (94.45)	1,546 (93.30)	1,497 (90.29)	1,464 (88.14)		
Yes	561 (8.46)	92 (5.55)	111 (6.70)	161 (9.71)	197 (11.86)		
Hypertension						746.02	**<0**.**0001**
No	5,106 (77.33)	1,567 (94.97)	1,392 (84.41)	1,200 (72.95)	947 (57.08)		
Yes	1,497 (22.67)	83 (5.03)	257 (15.59)	445 (27.05)	712 (42.92)		
Dyslipidemia						51.00	**<0**.**0001**
No	5,897 (90.67)	1,535 (94.23)	1,490 (91.75)	1,445 (89.31)	1,427 (87.39)		
Yes	607 (9.33)	94 (5.77)	134 (8.25)	173 (10.69)	206 (12.61)		
Diabetes						24.08	**<0**.**0001**
No	6,225 (94.73)	1,593 (96.72)	1,557 (95.05)	1,537 (94.12)	1,538 (93.04)		
Yes	346 (5.27)	54 (3.28)	81 (4.95)	96 (5.88)	115 (6.96)		
Cancer						6.19	0.10
No	6,550 (99.12)	1,630 (98.67)	1,642 (99.45)	1,637 (99.15)	1,641 (99.21)		
Yes	58 (0.88)	22 (1.33)	9 (0.55)	14 (0.85)	13 (0.79)		
Liver Disease						4.99	0.17
No	6,332 (96.14)	1,570 (95.27)	1,584 (96.41)	1,582 (96.23)	1,596 (96.67)		
Yes	254 (3.86)	78 (4.73)	59 (3.59)	62 (3.77)	55 (3.33)		
CLD						14.22	**<0**.**01**
No	6,035 (91.30)	1,534 (92.91)	1,517 (91.94)	1,504 (90.99)	1,480 (89.37)		
Yes	575 (8.70)	117 (7.09)	133 (8.06)	149 (9.01)	176 (10.63)		
Heart Disease						87.10	**<0**.**0001**
No	5,916 (89.65)	1,557 (94.31)	1,504 (91.32)	1,451 (87.99)	1,404 (84.99)		
Yes	683 (10.35)	94 (5.69)	143 (8.68)	198 (12.01)	248 (15.01)		
Stroke						11.64	**<0**.**01**
No	6,523 (98.64)	1,639 (99.09)	1,632 (98.91)	1,630 (98.73)	1,622 (97.83)		
Yes	90 (1.36)	15 (0.91)	18 (1.09)	21 (1.27)	36 (2.17)		
Kidney Disease						4.52	0.21
No	6,171 (93.64)	1,557 (94.65)	1,525 (92.87)	1,542 (93.51)	1,547 (93.53)		
Yes	419 (6.36)	88 (5.35)	117 (7.13)	107 (6.49)	107 (6.47)		
Stomach Disease						23.77	**<0**.**0001**
No	5,122 (77.50)	1,215 (73.68)	1,273 (77.25)	1,298 (78.48)	1,336 (80.58)		
Yes	1,487 (22.50)	434 (26.32)	375 (22.75)	356 (21.52)	322 (19.42)		
Arthritis						16.24	**<0**.**01**
No	4,443 (67.10)	1,170 (70.69)	1,117 (67.57)	1,083 (65.48)	1,073 (64.68)		
Yes	2,178 (32.90)	485 (29.31)	536 (32.43)	571 (34.52)	586 (35.32)		

The data presented in this table are derived from a comprehensive study involving 6,639 participants, categorized into four quartiles (Q1–Q4) based on certain criteria. The table provides detailed statistics on various physiological and demographic parameters, including but not limited to ePWV, age, sleeping time, weight, height, BMI, waist, grip, and more. The *p*-values indicate the statistical significance of differences among the quartiles for each variable, with a *p*-value < 0.05 suggesting a significant difference. Additionally, the table also presents information on the prevalence of sarcopenia, hypertension, dyslipidemia, and other health conditions across the quartiles.The data in the table are presented as mean ± standard deviation or number (percentage).

ePWV, estimated pulse wave velocity; BMI, body mass index; SMI, skeletal muscle index; CRP, C-reactive protein; HDL, high-density lipoprotein; LDL, low-density lipoprotein; IADL, instrumental activities of daily living, yes” indicates significant functional impairment, and “no” indicates no significant functional impairment; CLD, chronic lung disease; Q1–Q4, quartile 1 to quartile 4.

Bold values indicate statistical significance (*p* < 0.05).

### 3.2 Nested models

This study conducted an in-depth analysis of the relationship between muscle function indicators and arterial stiffness (represented by ePWV) using multiple regression models. Four models were employed: a crude model and three stepwise adjusted models to control for potential confounding factors.

Initially, the relationship between sarcopenia and continuous ePWV showed a significant positive correlation in the crude model. The *β* value was 1.35 (95% CI: 1.29, 1.41, *p* < 0.0001) in the crude model, but this significant crude association diminished in the adjusted models, suggesting confounding by factors such as age, which were controlled for in the subsequent analyses. In the quartile analysis, using Q1 as a reference, Q3 showed a correlation with sarcopenia, and a potential non-linear relationship with an initial increase followed by a decrease was observed from Q1 to Q4. Increasing ePWV was significantly associated with lower grip strength (*β* = −0.61, 95% CI: −0.74 to −0.48, *p* < 0.0001) in the crude model, but no significant association was observed in the adjusted models, indicating that the crude association was likely confounded by factors such as age. The quartile analysis results for grip strength were not statistically significant (*p* > 0.05).

Increasing ePWV was significantly associated with an increase in the time taken for the five-time chair stand test only in the crude model (*β* = 0.42, 95% CI: 0.37, 0.46, *p* < 0.0001). In the quartile analysis, Q3 showed *p* < 0.05 in Model 2 and Model 3.

Increasing ePWV was also significantly associated with a decrease in the skeletal muscle index in the unadjusted model (*β* = −0.05, 95% CI: −0.06, −0.03, *p* < 0.0001). This connection sustained its significance across all adjusted models, with the β coefficient rising to −0.01 (95% CI: −0.01, 0.00, *p* < 0.0001) in Model 3. Findings from the quartile analysis demonstrated that this relationship remained significant in all models (excluding Model 1), with a p trend of less than 0.01. All these results can be found in Table 2.

**Table 2:** OLS or logistic regression model on ePWV and sarcopenia, SMI, five times sit to stand test and grip.

Character	Crude model		Model 1		Model 2		Model 3	
	95%CI	*P*	95%CI	*P*	95%CI	*P*	95%CI	*P*
Sarcopenia ∼ePWV	1.35 (1.29, 1.41)	<0.0001	0.98 (0.89, 1.07)	0.61	0.99 (0.91, 1.09)	0.91	1 (0.91, 1.10)	0.98
Grip∼ePWV	−0.6 (−0.74, −0.46)	<0.0001	0.13 (−0.02, 0.29)	0.10	0.14 (−0.03, 0.30)	0.11	0.13 (−0.04, 0.30)	0.14
Five times sit to stand test ∼ePWV	0.42 (0.37, 0.46)	<0.0001	0.06 (−0.01, 0.13)	0.09	0.02 (−0.06, 0.09)	0.63	0.02 (−0.06, 0.09)	0.64
SMI∼ePWV	−0.05 (−0.06, −0.03)	<0.0001	0 (−0.01, 0.00)	<0.001	−0.01 (−0.01, 0.00)	<0.0001	−0.01 (−0.01, 0.00)	<0.0001
S2015∼ePWVQ								
Q1	Ref		Ref		Ref		Ref	
Q2	1.56 (1.19, 2.04)	0.001	1.18 (0.86, 1.63)	0.31	1.21 (0.87, 1.67)	0.26	1.21 (0.87, 1.68)	0.25
Q3	2.26 (1.75, 2.91)	<0.0001	1.42 (1.02, 1.98)	0.04	1.45 (1.03, 2.04)	0.03	1.46 (1.04, 2.06)	0.03
Q4	3.72 (2.92, 4.74)	<0.0001	1.16 (0.78, 1.73)	0.45	1.24 (0.82, 1.86)	0.31	1.25 (0.83, 1.89)	0.28
*p* for trend		<0.0001		0.5		0.35		0.36
Grip∼ePWVQ								
Q1	ref		ref		ref		ref	
Q2	0.55 (−0.10, 1.19)	0.10	0.47 (−0.01, 0.96)	0.06	0.47 (−0.03, 0.97)	0.07	0.46 (−0.04, 0.96)	0.07
Q3	−0.4 (−1.04, 0.25)	0.23	0.51 (−0.02, 1.05)	0.06	0.53 (−0.03, 1.09)	0.06	0.52 (−0.04, 1.07)	0.07
Q4	−2.07 (−2.72, −1.43)	<0.0001	0.6 (−0.06, 1.25)	0.07	0.63 (−0.06, 1.33)	0.08	0.62 (−0.08, 1.31)	0.08
*p* for trend		<0.0001		0.09		0.07		0.08
Five times sit to stand test ∼ePWVQ								
Q1	Ref		Ref		Ref		Ref	
Q2	0.47 (0.25, 0.69)	<0.0001	0.06 (−0.16, 0.28)	0.59	0.03 (−0.20, 0.25)	0.81	0.02 (−0.20, 0.24)	0.87
Q3	0.66 (0.44, 0.88)	<0.0001	−0.2 (−0.44, 0.04)	0.10	−0.3 (−0.55, −0.05)	0.02	−0.31 (−0.55, −0.06)	0.01
Q4	1.7 (1.49, 1.92)	<0.0001	0.13 (−0.17, 0.42)	0.40	−0.04 (−0.35, 0.27)	0.79	−0.04 (−0.35, 0.26)	0.78
*p* for trend		<0.0001		0.94		0.28		0.29
SMI∼ePWVQ								
Q1	Ref		Ref		Ref		Ref	
Q2	0.18 (0.11, 0.25)	<0.0001	0 (−0.01, 0.01)	0.59	0 (−0.01, 0.01)	0.99	0 (−0.01, 0.01)	0.98
Q3	0.13 (0.06, 0.20)	<0.001	0 (−0.01, 0.01)	0.88	0 (−0.01, 0.01)	0.52	0 (−0.01, 0.01)	0.52
Q4	−0.11 (−0.18, −0.03)	0.004	−0.01 (−0.02, 0.00)	0.07	−0.02 (−0.03, 0.00)	0.01	−0.02 (−0.03, −0.01)	0.01
*p* for trend (character2integer)		0.001		0.12		0.01		0.01

ePWV.

Crudel model: ePWV.

Model 1: ePWV, age, sex, BMI, marital status, education, Residence, night hour.

Model 2: ePWV, age, sex, BMI, marital status, education, Residence, night hour, smoke, Drink, IADL, DM, CLD, Hypertension, Cancer, Liver Disease, Kidney Disease, Stomach Disease, Dyslipidemia, Arthritis.

Model 3: ePWV, age, sex, BMI, marital status, education, Residence, night hour, smoke, Drink, IADL, DM, CLD, Hypertension, Cancer, Liver Disease, Kidney Disease, Stomach Disease, Dyslipidemia, Arthritis, CRP, Creatinine, HDL, LDL.

In the initial analysis of the relationship between ePWV and sarcopenia, a significant positive crude association was observed (*β* = 1.35 (95% CI: 1.29, 1.41, *p* < 0.0001). However, after adjusting for a series of covariates including age, gender, BMI, and various health-related behaviors and chronic conditions in the subsequent models, this association diminished and became non-significant. This clearly indicates that there are confounding factors at play.

### 3.3 RCS analysis

**Figure 2:**
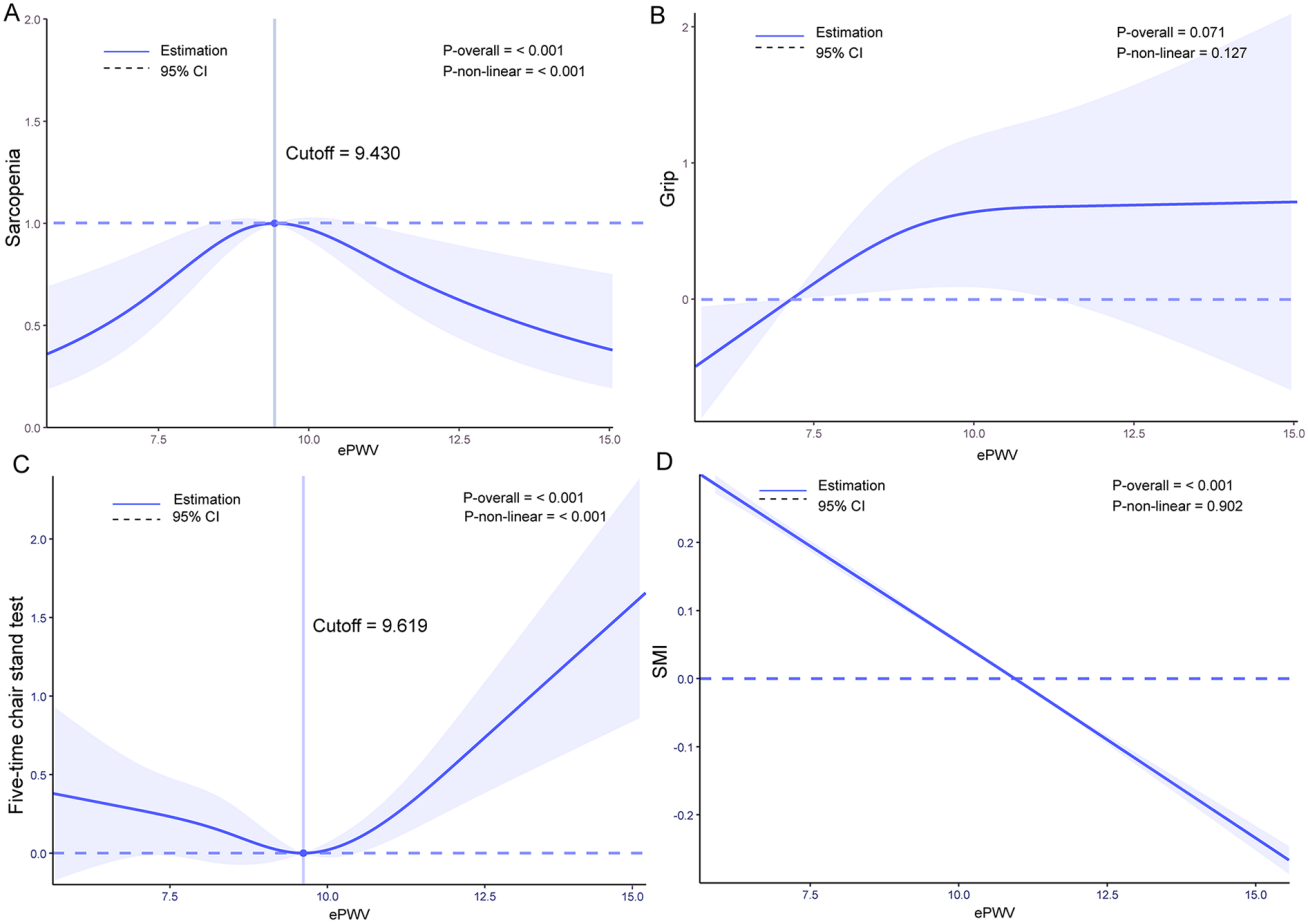
Restricted cubic spline (RCS) analysis results: **(A)** relationship between estimated pulse wave velocity (ePWV) and sarcopenia incidence, **(B)** relationship between ePWV and grip strength, **(C)** relationship between ePWV and five times Sit to stand test results, **(D)** relationship between ePWV and skeletal muscle Index (SMI).

We conducted Restricted Cubic Spline (RCS) analysis based on Model 3 mentioned above. For the relationship between ePWV and the incidence of sarcopenia, we found that as ePWV increased, the incidence of sarcopenia first increased and then decreased. This overall trend was statistically significant (P overall <0.001) and showed a non-linear relationship (P-non-linear <0.001). The turning point was at ePWV = 9.430 m/s, where the probability of sarcopenia occurrence was highest. No linear or non-linear relationship was found between ePWV and grip strength. For the relationship between ePWV and the five-time chair stand test, we discovered that as ePWV increased, the time taken for the five-time chair stand test first decreased and then increased. The distribution before ePWV = 9.61 m/s showed no significant difference, but after ePWV = 9.619, the time spent increased sharply. Both the overall trend and non-linearity were significant with *P* values < 0.001. The relationship between ePWV and SMI (Skeletal Muscle Index) showed a trend of decreasing SMI as ePWV increased (P overall <0.001). The results of these analyses can be seen in Figure 2.

### 3.4 Subgroup analysis

**Figure 3:**
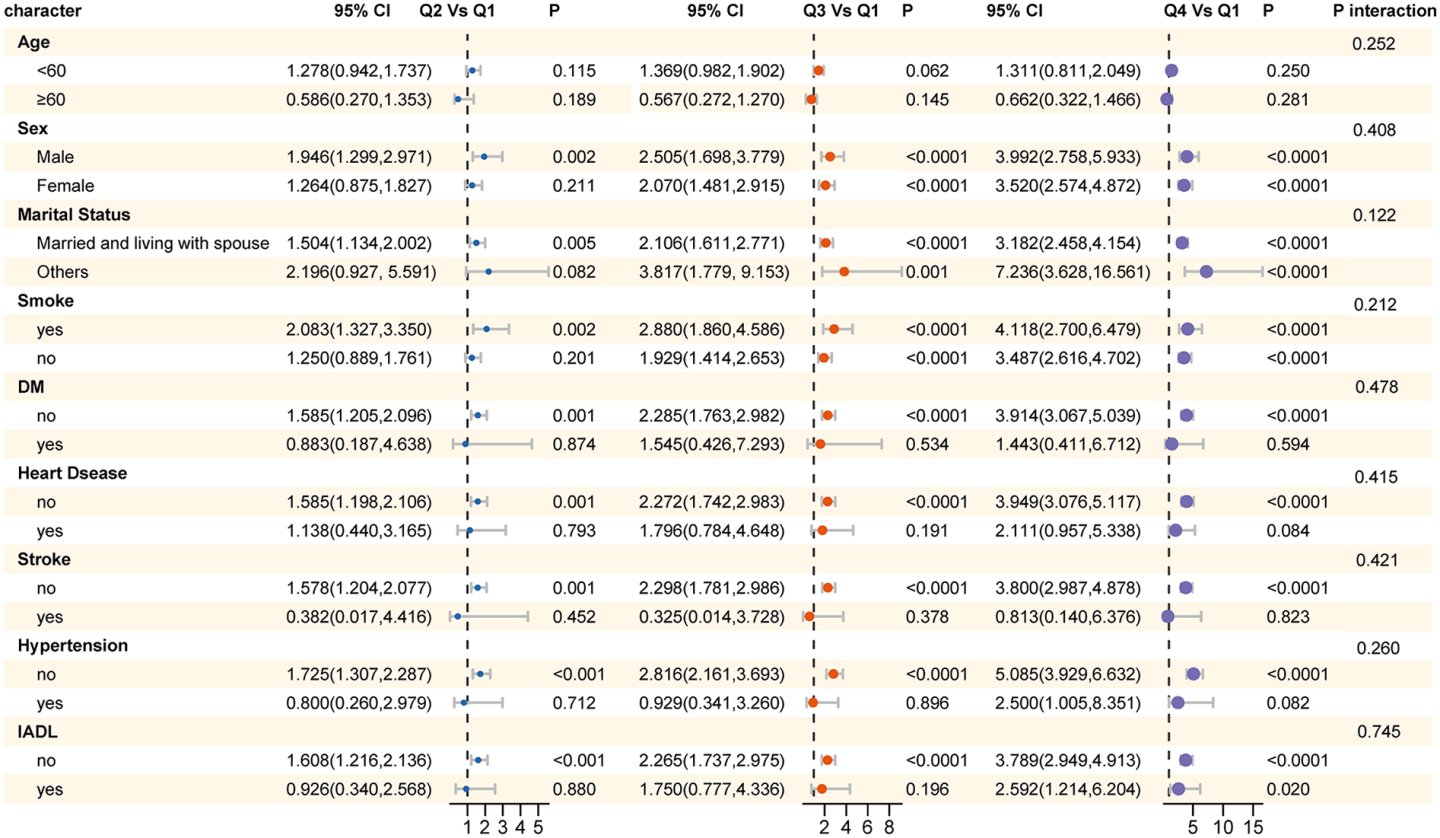
Results of subgroup analysis for the association between ePWV and sarcopenia across different population subgroups.

Subgroup analyses were conducted using logistic regression models to examine the relationship between ePWV and sarcopenia across various population subgroups. These subgroups were defined based on age (<60 years and >60 years), gender, marital status, smoking status, diabetes, heart disease, stroke, hypertension, and disability in daily activities. The results revealed that the risk of sarcopenia was higher in males, individuals with marital statuses other than married, and smokers. Importantly, no statistically significant interaction effects were observed across any of the subgroups, with all P interaction values exceeding 0.05. These findings suggest that the relationship between ePWV and sarcopenia remains consistent across different demographic and health-related subgroups. Detailed results of these analyses are presented in Figure 3.

## 4 Discussion

This research utilized data from the China Health and Retirement Longitudinal Study (CHARLS) to investigate the connection between estimated pulse wave velocity (ePWV) and sarcopenia. Employing logistic regression models alongside restricted cubic spline (RCS) analysis, a non-linear pattern was identified in the incidence of sarcopenia in relation to increasing ePWV, with the risk peaking at a certain ePWV threshold (9.430 m/s). Additionally, we discovered inverse relationships between ePWV and both muscle strength as well as the skeletal muscle index (SMI), which further corroborates the association between arterial stiffness and the reduction of muscle mass and functionality. In subgroup analyses, an increase in the risk of sarcopenia was consistently observed with elevated ePWV across various populations.

Compared to the conventional method of assessing arterial stiffness, which is PWV, ePWV derived from age and blood pressure offers advantages such as being time-efficient, easy to use, and cost-effective. Research indicates that ePWV maintains strong consistency and is valuable for predicting cardiovascular risk in Western populations (13, 27, 28). Numerous earlier cross-sectional investigations have identified links between arterial stiffness and skeletal muscle mass (20, 29–31). For instance, Abbatecola et al. (20) carried out a significant cohort study that determined a negative correlation between pulse wave velocity and skeletal muscle mass in Americans aged 70–90 years. Additionally, Sampaio et al. (29) found a substantial relationship between arterial stiffness, measured via the cardio-ankle vascular index (CAVI), and low muscle mass index among elderly Japanese living in the community.

Furthermore, Tanaka et al. (30) demonstrated that among 97 postmenopausal women with type 2 diabetes, reduced muscle mass and higher visceral fat were independently linked to increased arterial stiffness. Ochi et al. discovered that baPWV served as an independent risk factor for sarcopenia in men when controlling for various factors, including age, height, physical inactivity, and levels of free testosterone (31). In our research, we utilized ePWV to evaluate arterial stiffness and found a significant negative correlation between ePWV and SMI after accounting for all confounding variables, reinforcing the idea of an inverse relationship between pulse wave velocity and skeletal muscle mass.

Sarcopenia is influenced not just by muscle mass, but also by factors such as muscle strength and gait speed. According to Gonzales’ research, when controlling for variables like age, body mass index, waist circumference, and systolic blood pressure, a negative correlation was found between gait performance and both carotid-femoral pulse wave velocity and carotid stiffness index (32). A meta-analysis, which incorporated 6 cross-sectional studies involving 5,476 participants, indicated that the standardized mean difference in pulse wave velocity was greater among individuals with sarcopenia than in their non-sarcopenic counterparts (33). Furthermore, the investigation by Jiang et al. proposed that elevated brachial-ankle pulse wave velocity (baPWV) may serve as a partial mediator in the relationship between age and sarcopenia among individuals diagnosed with type 2 diabetes (34). This cohort study expanded on prior work by examining the connection between ePWV, a marker of arterial stiffness, and all aspects of sarcopenia — which encompasses skeletal muscle mass, muscle strength, and physical performance — alongside various muscle function indicators. Our analysis revealed that in the unadjusted model, increased ePWV could potentially correlate with a greater occurrence of sarcopenia, thus reinforcing the conclusions drawn from earlier studies.

Multiple cross - sectional studies have shown that lower lean muscle mass and sarcopenia are associated with increased arterial stiffness and arteriolosclerosis (21, 35, 36). Although the specific causal mechanisms underlying the connection between arterial dysfunction and the decrease in muscle mass and function still need to be more deeply elucidated, numerous theories have emerged at present (36, 37). Among them, chronic inflammation, oxidative stress, insulin resistance, and blood flow obstruction caused by endothelial dysfunction and calcification of the skeletal muscle vasculature are all regarded as possible causes (37).

The link between arterial stiffness, a key indicator of cardiovascular system aging (38), and sarcopenia might be explained by inflammation. Studies have shown that elevations in IL-6 and CRP may particularly play a role in the inflammatory link between sarcopenia and cardiovascular risk (39). As age increases, the inflammatory response triggered by aging not only exacerbates arterial stiffness but may also promote the production of more inflammatory factors. High levels of these inflammatory mediators may negatively impact the maintenance and repair processes of muscle cells, thereby promoting the breakdown of muscle tissue (36). Furthermore, microvascular dysfunction may be another important mechanism connecting arterial stiffness with sarcopenia. During the aging process, the gradual increase in arterial stiffness becomes particularly closely intertwined with the microvascular system, potentially leading to local ischemia and microvascular dysfunction (40). This damage may further impair the microvascular function of skeletal muscle (41), reducing its oxidative capacity and affecting nutrient delivery, ultimately potentially leading to sarcopenia (42). Although our current understanding of these mechanisms is not complete, future research will help to explore in depth the complex relationship between arterial stiffness and sarcopenia and its potential biological mechanisms.

Our RCS analysis unexpectedly revealed a non-linear relationship between ePWV and the incidence of sarcopenia. This non-linear relationship may be attributed to complex biological interactions between ePWV and sarcopenia. At lower ePWV levels, increased arterial stiffness might exacerbate hemodynamic changes in muscle tissue, thereby increasing the risk of sarcopenia. However, when ePWV reaches a certain threshold, it may trigger compensatory mechanisms such as vascular remodeling or blood flow redistribution (43), which could potentially mitigate the progression of sarcopenia to some extent. Additionally, this non-linear relationship may be related to the multifactorial etiology of sarcopenia. The development of sarcopenia is likely influenced by various factors, including age, gender, nutritional status, and activity level (8), which may have differential effects on sarcopenia incidence at varying ePWV levels.

Subgroup analysis revealed a consistent trend across all subgroups: as ePWV values increased, the risk of sarcopenia also increased. This finding further suggests a potential dose-response relationship between arterial stiffness and muscle health. The lack of statistical interaction between subgroups indicates that the relationship between ePWV and sarcopenia does not significantly differ across different subgroups. This may imply that our model is relatively stable, meaning the impact of arterial stiffness on the risk of sarcopenia is relatively uniform across the studied population. However, it does not negate the potential importance of other factors that may influence this relationship. For instance, lifestyle and chronic diseases may also play a role in the development of sarcopenia (44).

This cohort study, through long-term follow-up, reveals a potential link between changes in ePWV and the incidence of sarcopenia. To our knowledge, this is the first study to explore the relationship between ePWV and sarcopenia, and the cohort study design helps to investigate causal relationships. The results of this study indicate that after adjusting for confounding factors such as age, BMI, and comorbidities, the relationship between ePWV and the occurrence of sarcopenia no longer has statistical significance (*P* > 0.05). However, it is worth noting that there is still a strong linear relationship between ePWV and SMI, a key index related to sarcopenia. Moreover, regardless of whether adjustment has been made, compared with the Q1 group of ePWV, the occurrence of sarcopenia in the Q3 group shows a significant correlation. It is undeniable that confounding factors such as age may have played a non—negligible role. But in the complete restricted cubic spline (RCS) analysis, we clearly observed a non—linear relationship between ePWV and the incidence of sarcopenia, which strongly indicates that ePWV still has potential predictive value for the occurrence of sarcopenia. Due to the use of a single—center database and a relatively small sample size, this study inevitably has certain limitations. Also, although we controlled for multiple confounding factors, some unmeasured variables, such as environmental exposures and genetic factors, may influence the outcomes. Furthermore, differences in ePWV measurement methods and sarcopenia diagnostic criteria across research centers may interfere with the consistency of results. However, it cannot be ignored that this non—linear relationship objectively exists in the middle-aged and elderly population in China. This non-linear relationship could potentially guide the development of personalized prevention and intervention strategies based on an individual's ePWV level.

Looking ahead, this study lays the foundation for further exploration of ePWV's potential as an early diagnostic indicator for sarcopenia. Future research should expand the sample size and cover a wider range of age and ethnic groups to enhance the universality of the findings. Additionally, we recommend adopting a multi-center, multinational research design to mitigate the limitations of a single research center. Future research could also focus on developing more refined models that incorporate ePWV along with other relevant factors to enhance the predictive accuracy. For example, further investigations could explore the combination of ePWV with muscle strength and physical performance measures in a multivariate model to better predict the risk of developing sarcopenia. Moreover, in-depth research into the biological mechanisms between ePWV and sarcopenia will contribute to the development of new prevention and treatment strategies, providing more precise interventions for health management in elderly populations.

## 5 Conclusion

The results of this research suggest that variations in ePWV may be associated with the likelihood of developing sarcopenia in individuals aged 45 and over in the Chinese population. A non-linear association was noted between ePWV, which serves as a marker of arterial stiffness, and the risk of sarcopenia. These findings offer a new viewpoint on the relationship between arterial stiffness and sarcopenia, suggesting that ePWV could potentially serve as a predictive indicator for sarcopenia. Nonetheless, the specific mechanisms that link ePWV to sarcopenia necessitate additional exploration.

## Data Availability

The raw data supporting the conclusions of this article will be made available by the authors, without undue reservation.
